# Rac1 as a Target to Treat Dysfunctions and Cancer of the Bladder

**DOI:** 10.3390/biomedicines10061357

**Published:** 2022-06-08

**Authors:** Vincent Sauzeau, Julien Beignet, Christian Bailly

**Affiliations:** 1 Université de Nantes, CHU Nantes, CNRS, INSERM, Institut du Thorax, 44202 Nantes, France; vincent.sauzeau@inserm.fr; 2 SATT Ouest Valorisation, 30 Boulevard Vincent Gâche, CS 70211, CEDEX, 44202 Nantes, France; julien.beignet@ouest-valorisation.fr; 3 OncoWitan, Scientific Consulting Office, 59290 Lille (Wasquehal), France

**Keywords:** bladder cancer, Rho GTPase, bladder dysfunction, Rac inhibitors, metastasis, overactive bladder, Rac1 protein

## Abstract

Bladder pathologies, very common in the aged population, have a considerable negative impact on quality of life. Novel targets are needed to design drugs and combinations to treat diseases such as overactive bladder and bladder cancers. A promising new target is the ubiquitous Rho GTPase Rac1, frequently dysregulated and overexpressed in bladder pathologies. We have analyzed the roles of Rac1 in different bladder pathologies, including bacterial infections, diabetes-induced bladder dysfunctions and bladder cancers. The contribution of the Rac1 protein to tumorigenesis, tumor progression, epithelial-mesenchymal transition of bladder cancer cells and their metastasis has been analyzed. Small molecules selectively targeting Rac1 have been discovered or designed, and two of them—NSC23766 and EHT 1864—have revealed activities against bladder cancer. Their mode of interaction with Rac1, at the GTP binding site or the guanine nucleotide exchange factors (GEF) interaction site, is discussed. Our analysis underlines the possibility of targeting Rac1 with small molecules with the objective to combat bladder dysfunctions and to reduce lower urinary tract symptoms. Finally, the interest of a Rac1 inhibitor to treat advanced chemoresistance prostate cancer, while reducing the risk of associated bladder dysfunction, is discussed. There is hope for a better management of bladder pathologies via Rac1-targeted approaches.

## 1. Introduction

**Figure 1:**
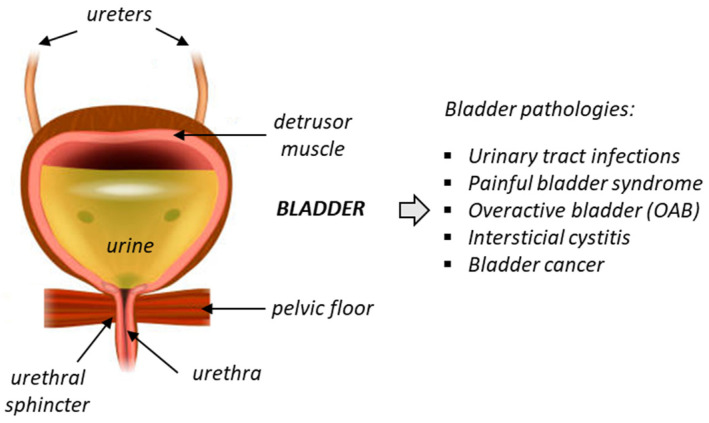
Anatomy of the bladder and the main associated pathologies.

Chronic bladder diseases are frequent and have a significant impact on quality of life. Unfortunately, the current treatment options for these diseases are limited and often unsatisfactory. Deficiency of bladder functions generally lead to failure to store urine or empty the bladder, and these defects can cause a variety of problems, such as incontinence, frequent urination, urinary retention, and bladder pain. One of the most common urinary pathologies is idiopathic overactive bladder (OAB), frequently diagnosed in elderly populations (Figure 1). OAB is a condition where the bladder muscle contracts involuntarily. It is characterized by storage-related lower urinary tract symptoms (LUTS), such as urgency incontinence and nocturia. The prevalence of OAB is high in developed countries, estimated to 10–16% of the population and increasing with age [1]. The economic impact of the disease is huge, with an estimated total national cost of OAB reaching USD 82.6 billion in 2020 for the US only [2,3]. The pathology affects both sexes, although the proportion of women suffering from OAB (notably wet OAB associated with urgency urinary incontinence) is apparently higher than in men [4].

The pharmacotherapy of OAB relies essentially on the use of two β_3_-adrenergic agonists: vibegron (*Gemtesa^®^*, FDA-approved in 2020) and mirabegron (*Myrbetriq^®^*, FDA-approved in 2012) with a comparable limited efficacy, even if vibegron, with less side effect on blood pressure, is often preferred to mirabegron [5,6,7,8]. A few other options are available, including the use of botulinum toxin A, neuromodulation with implantable devices, and laser treatment but their efficacy is limited [9,10,11]. The search for novel treatment modalities and drugs continues. New compounds with β_3_-adrenergic agonistic properties are regularly proposed [12,13,14], as well as novel molecular targets, such as antagonists of TRPM8 (transient receptor potential melastatin 8) channels [15] or other TRPM-type channels [16].

**Figure 2:**
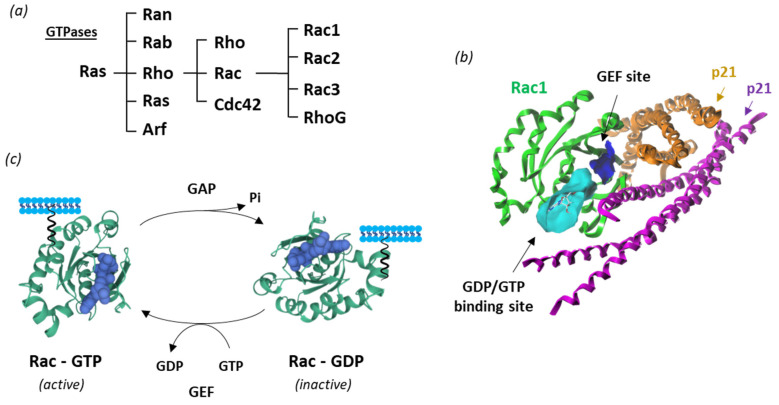
Rac1 structure and activity. (**a**) Classification of Ras GTPases. (**b**) A molecular model of Rac1 interacting with p21 (arfaptin) (PDB: 1I4D) with the GTP/GDP (cyan) and GEF (blue) binding sites illustrated [21] (**c**) Cycle of Rac1 activation. The GTPase cycles between an active GTP-bound state and an inactive GDP-bound state. Guanine nucleotide exchange factors (GEF) turn on signaling by catalyzing the exchange from G-protein-bound GDP to GTP, whereas GTPase activating proteins (GAP) terminate signaling by inducing GTP hydrolysis. GEF and GAP regulate the activity of Rac1 and other small guanine nucleotide-binding (G) proteins to control cellular functions.

New therapeutic targets for OAB at the levels of the bladder urothelium, detrusor muscle, spinal cord and brain have been proposed [17,18]. This is the case of Rho guanosine triphosphatases (Rho GTPases), which correspond to a family of GTP-binding proteins largely implicated in regulating actin cytoskeleton dynamics and several cellular signaling pathways. There are 20 Rho GTPases divided into eight subfamilies, based on their structure and mechanism of enzymatic activity [19,20]. Among them, the Rac subgroup includes the four members, designated Rac1-3 and RhoG, involved in a variety of mechanisms and human pathologies (Figure 2).

Dysregulations of Rac signaling have been reported in atherosclerosis [22], neurodevelopmental disorders [23], rheumatic diseases [24], pulmonary hypertension [25] and different types of cancers, including urothelial carcinoma [26,27]. Protein Rac1 (RAS-related C3 botulinum toxin substrate (1) is considered a prime target to combat a variety of solid tumors and certain onco-hematological malignancies. The role and targeting of Rac1 in cancers have been debated in recent years [28,29,30,31], but the specific implication of Rac1 in OAB and bladder cancer has not been assessed.

Here, we provide an analysis of the role of Rac1 in major bladder pathologies and a pharmacological approach to target the Rac1 pathway. Specifically, we have analyzed the role of Rac1 in bacterial infections of the bladder, in diabetes-induced bladder dysfunctions and in the various steps of bladder cancer (tumorigenesis, tumor progression, metastasis). In the three situations, Rac1 plays significant roles, and the targeting of the protein could be a convenient approach to reduce the progression of those diseases. Rac1 inhibitors could be useful to combat these different pathologies, in particular, to tackle OAB associated with cancer.

## 2. Rac1 Structure and Function

Like many other GTPases, Rac1 switches between an inactive GDP-bound and an active GTP-bound state during signal transduction [32] (Figure 2c). The protein is involved in a wide range of cellular and physiological processes via a multiplicity of protein partners, among which a variety of guanine nucleotide exchange factors (GEFs) and GTPase-activating proteins (GAPs) essential to control Rac1 activity [33]. In addition, a diversity of effector proteins can modulate Rac1 function, such as the serine-threonine kinases PAK1 (p21-activated kinase 1), MLK-1-3 (mixed-lineage kinases), p70 S6 kinase, CaMKII and many other kinases [20]. The local availability of GTP in cells plays a role in the control of Rac1 activity [34]. The expression and subcellular localization of Rac1 is also regulated at the post-transcriptional level via structural modifications, including phosphorylation, ubiquitination, adenylylation, and lipidation (prenylation, geranylgeranylation, palmitoylation) [35]. The lipid anchor is positioned in a hypervariable region, distant from the guanine nucleotide-binding domain, but contributing importantly to the interaction with effectors [36]. The protein is generally attached to the plasma membrane, but it can also be found in the nucleus and/or mitochondria [37]). At the membrane level, Rac1 can form nanoclusters acting as lipid-based signaling platforms [38].

The GDP/GTP loading status and cycling rate of Rac1 determine the protein activity. The nucleotide cycling process is impacted by the intrinsic conformational flexibility of the protein and the Mg^2+^ abundance [39]. The level of expression of the protein can vary significantly. An epigenetic downregulation of Rac1 has been reported in patients suffering from depression [40]. Conversely, Rac1 is often overexpressed and hyperactivated in cancers, notably in breast, colon, skin (melanoma), liver and lung cancers [31,41,42,43]. Moreover, Rac1 gain-of-function mutations have been identified in recent years, such as the two somatic mutations Rac1^P29S^ and Rac1^A159V^, respectively detected in melanoma and in head-and-neck cancers [44,45,46], and occasionally observed in colon, thyroid, and lung cancers [47]. These variants represent fast cycling mutants that contribute to expand tumor phenotypes and confer resistance to targeted therapies. There exists also an alternatively spliced isoform designated Rac1b, with versatile functions, generally involved in tumor progression, but occasionally described as being engaged in the blockade of tumors [48,49]. To our knowledge, neither the fast-cycling oncogenic mutant enzymes nor the spliced variant Rac1b have been reported in bladder cancer or bladder pathologies.

## 3. Rac1 in Non-Cancerous Bladder Pathologies

### 3.1. Rac1 and Bacterial Infections of the Bladder

**Figure 3:**
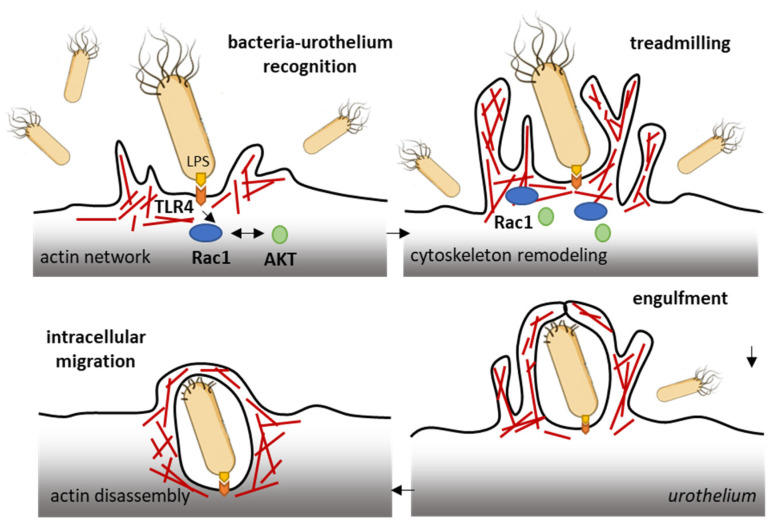
Rac1 plays a role in the invasion of bladder epithelial cells by type 1 fimbriated *E. coli. Bacterial* lipopolysaccharides activate the TLR4/Rac1/Akt signaling pathway to enter cells vascular smooth muscle cells and colonize the bladder tissue [54]. The Rac1 GTPase-mediated contributes to actin cytoskeleton remodeling and regulation of actin filaments.

The ubiquitous Rho GTPase Rac1 plays key roles in the regulation of the cytoskeleton dynamic and cell motility in general. The protein is associated with the formation of protrusions at leading edge of migrating cells (lamellipodia, filopodia), whatever the cell type. As such, Rac1, actively participates to control cellular proliferation and cell mobility. It also plays a role in bacterial attachment to host cells and infections, notably in bladder infections caused by uropathogenic *Escherichia coli* [50]. Urinary tract infection (UTI) produced by uropathogenic *E. coli* (UPEC) promotes the sensitization of bladder afferent sensory neurons and the virulence factors produced by those bacteria contribute to the sensitization of bladder afferents in UTI [51]. The uroepithelial invasion by the bacteria occurs through lipid rafts, and Rac1 associated with caveolin-1 in those rafts is required for the bacterial invasion [52]. Rac1 activation enhances the accumulation of actin filaments at sites of bacterial entry (Figure 3). The use of bladder epithelial cells overexpressing constitutively activated Rac1, or conversely, cells with the dominant negative form, has clearly demonstrated that Rac1 activation is essential to the invasion of bladder epithelial cells by type 1 fimbriated *E. coli.* Moreover, the inhibition of Rac-1 activation via a Toll-like receptor 4 (TLR4)-mediated mechanism was found to suppress bacterial invasion [53]. In fact, bacterial lipopolysaccharides engage the TLR4/Rac1/Akt signaling pathway to enter cells and mediate the proliferation of vascular smooth muscle cells [54]. Once in the cells, the bacteria produce a toxin CNF1 (cytotoxic necrotizing factor type (1), which constitutively activates different Rho GTPases, including Rac1 critical to phagocytosis, to promote further infection [55]. The key role of Rac1 in the invasion of bladder epithelial cells by uropathogenic bacteria suggests that a negative regulation of Rac1 can be an option to reduce and combat infections of the urinary bladder. This can be achieved directly with Rac1-targeting small molecules (discussed below) or indirectly with compounds interfering with Rac1-mediated actin polymerization, as shown with the dietary flavonoid luteolin, for example [56].

Rac1 is used by different types of microbial organisms to enter cells. This is also the case for the *Mycobacterium bovis* Bacille Calmette–Guerin (BCG) strain, which is used as a vaccine for tuberculosis. The BCG infection of primary airway epithelial cells has been shown to induce Rac1 up-regulation and to cause actin redistribution [57]. In bladder cancer cells, the entry of the BCG was found to rely on the expression of Rac1 and its effector kinase Pak1 (as well as Cdc42) via a process of micropinocytosis [58]. A BCG-induced enhanced expression of Rac1 has been reported in a study with infected macrophages, both in vitro and in vivo. The mycobacteria activated the p38K/JNK/b1-integrin/Rac1 signaling cascade in the frame of the infection [59].

The treatment of recurrent urinary tract infections usually relies on the use of intravenous antibiotic therapy (which can lead to complications due to allergy or drug-resistance). Repeated intravesical drug delivery is also possible, but it is more challenging [60]. The efficacy is these treatments is suboptimal at present. There is a need for new therapeutic options, new drugs and novel approaches in general to address the pathophysiology of the disease [61].

### 3.2. Rac1 and Diabetes-Induced Bladder Dysfunctions

Urinary bladder dysfunction is a complication in diabetes mellitus (DM) [62]. Diabetes causes bladder remodeling leading to uropathy in a mulitfactorial way, with neurogenic and myogenic detrusor overactivity and changes in transmitter regulation leading to a hyper-excitability of the detrusor [63]. DM is also a risk factor for bladder cancer prognosis and outcome [64]. Diabetic cystopathy (urinary disturbances) is one of the most common complications of diabetes mellitus [65]. The pathophysiology of the disease is complex and multifactorial, but it seems clear that Rac1 plays a role in the inflammatory mechanism, via binding to and the activation of the NOD-like receptor protein 3 (NLRP3) inflammasome. Indeed, under hyperglycemia conditions, Rac1 can promote NLRP3 inflammasome activation and induces cell damage [66]. The oxidative stress that occurs in the bladder of diabetic subjects causes oxidative damage to the urothelial and smooth muscle cells. A markedly enhanced expression of Pak1 (RAC1/p21 activated kinase 1) has been observed in the smooth muscle of diabetic mouse bladders versus the control group [67]. In a rat model of streptozotocin-induced diabetic bladder, an increased expression of Rac1 has been observed by immunohistochemistry. The Rac1 immunoreactivity was found to increase significantly in all the layers of the bladder tissue (epithelium, lamina propria, and tunica muscularis) for the diabetic group compared to the control group [68]. This study is important because it also showed that a Rac1 inhibitor (NSC23766) can inhibit the contractile responses of the bladder detrusor smooth muscle. This pharmacological aspect is discussed further below.

Interestingly, the expression of Rac1 in bladder tissue is increased not only due to the diabetes context, but it is also enhanced mechanically through the induced and cyclic hydrodynamic pressure exerted on bladder smooth muscle cells [69]. The expression of both Rac1 and phospho-Rac1 was found to be increased when a hydrodynamic pressure was mechanically applied onto human bladder smooth muscle cells. The expression of Rac1 downstream effectors, such as phospho-MEK1/2 and ERK-1/2, was also increased, and the effects were abrogated when cells were treated with a small molecule Rac1 inhibitor (NSC23766) or a Rac1 siRNA [69]. Rac1 seems to play an important role in the proliferation and response of bladder smooth muscle cells to hydrodynamic pressure. The data suggest that, in this situation, the use of Rac1 inhibitors could permit a reduction in bladder dysfunctions.

Another line of evidence showing that Rac1 plays an important role and is required for active contraction in smooth muscle comes from experiments using a conditional Rac1 knockout mouse strain. In this case, the loss of about 50% of Rac1 protein in the urinary bladder resulted in a significant decrease in the contractile responses to different agonists, without causing a remodeling of the vessels in the bladder tissue [70]. Similar effects were obtained using Rac1 inhibitors, as discussed below. In a recent study, the silencing of Rac1 expression in human bladder smooth muscle cells was found to reduce cell viability by 50–70% after 48 h and to increase the percentage of cells in (early/late) apoptosis compared to wild-type cells. The effects were associated with alterations in actin organization [71].

There are currently multiple pharmacological options to treat diabetes-induced bladder dysfunctions, notably using α_1_-adrenoceptor and muscarinic receptor antagonists, β_3_-adrenoceptor agonists and phosphodiesterase type 5 inhibitors [72]. However, here also, newer treatments and drugs are needed to improve long-term efficacy.

## 4. Rac1 in Bladder Cancer

The *Rac1* gene, like other Rho-related genes, is frequently overexpressed in urothelial cell carcinoma, and the altered expression of the corresponding proteins plays an important role in the genesis and progression of cancers of the urinary bladder [73,74]. The gene overexpression and alterations not only concern *Rac1* but also the associated regulatory elements, such as kinases PAK1 and PAK4 (P21 activated kinase 1/4), which are amplified and/or overexpressed in muscle-invasive bladder carcinomas [75,76,77]. A moderate or strong positive expression of both Rac1 and PAK1 are considered independent factors for shortened disease-specific survival time in patients with upper urinary tract urothelial carcinoma [78]. Numerous studies have reported alterations of Rac1 expression and function in bladder cancer, and the expressed protein has been associated with a variety of functional alterations. For the sake of clarity, we can refer to four categories of effects, briefly discussed in turn hereafter.

### 4.1. Rac1 in Bladder Tumorigenesis

**Figure 4:**
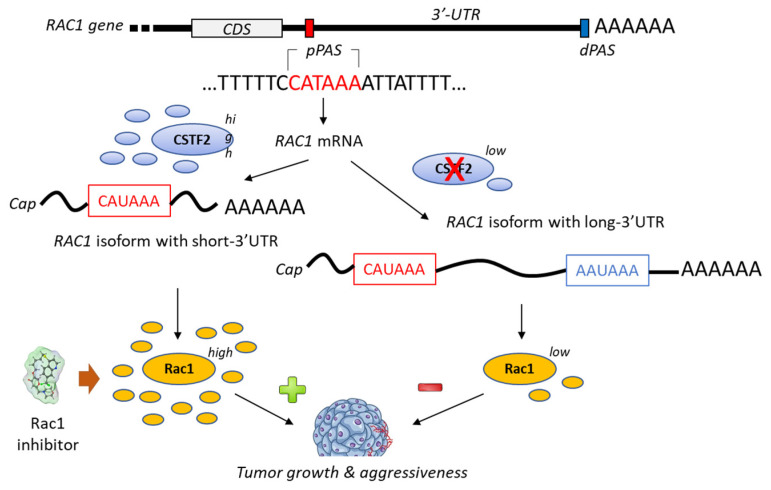
Proposed mechanism for the generation of short-3′-UTR (3′-untranslated region) isoform of *Rac1* in the presence of a high level of the cleavage/polyadenylation factor CSTF2 (cleavage stimulation factor (2). The *RAC1* short-3′UTR isoform has oncogenic functions and increases aggressiveness of cancer cells from urothelial bladder carcinoma UBC). The dual high expression of CSTF2 and Rac1 with short 3′-UTR predicts worse prognosis for UBC patients [79]. The proximal and distal polyadenylation sites (pPAS, dPAS) are located within the terminal exon. CDS, protein-coding sequence.

A bioinformatic analysis of mRNA from patients with urothelial carcinoma of the bladder has revealed the presence of a shorter 3’-UTR (3′-untranslated region) isoform of Rac1 and this specific isoform was associated with an upregulation of Rac1 protein expression (Figure 4). The formation of this isoform was apparently mediated by the recruitment of the cleavage stimulation factor 2 (CSTF2) at a polyadenylation site of *Rac1*, thereby reducing the recruitment of two transcription factors (AFF1 and AFF4), thus causing defects in elongation. The short 3’UTR isoform of *Rac1* apparently plays an essential oncogenic role in the pathogenesis of bladder cancer [79]. The enhanced expression of Rac1 is certainly not the sole key element contributing to bladder carcinogenesis; the modulation of the full Rho-GTPase axis has been implicated in bladder cancer tumorigenesis [80]. Rac1 plays a role in the carcinogenesis of various cancers, including bladder cancer but also hepatocarcinoma, breast cancer, non-small-cell-lung cancers and others [81,82].

### 4.2. Rac1 in Bladder Cancer Cells Proliferation and Tumor Progression

**Figure 5:**
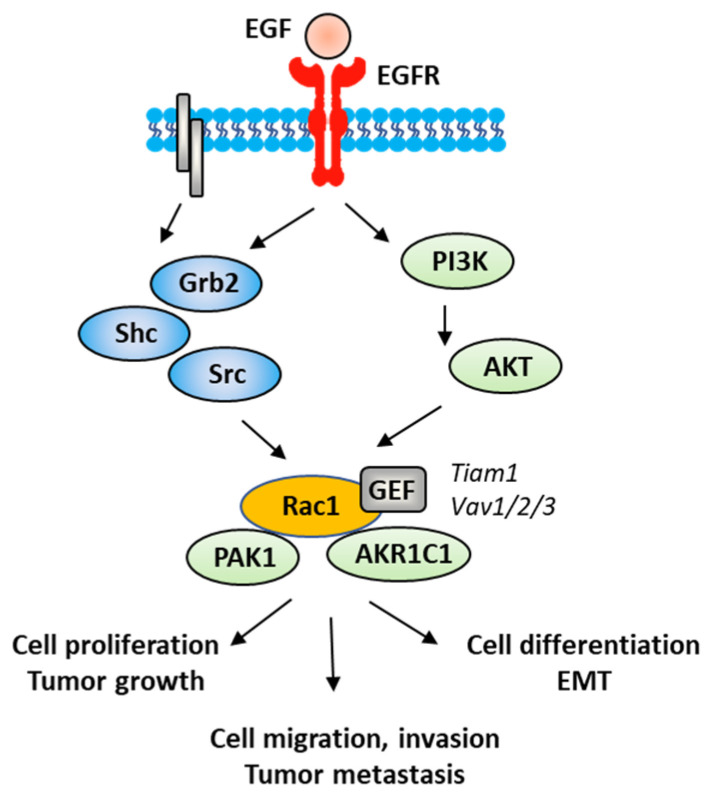
A signaling network between EGFR and Rac1, in vascular smooth muscle cells with various signaling effectors implicated.

The Rac1 protein is one of the elements that contributes to the proliferation and dissemination of bladder cancer cells. The Rac1 axis is a regulatory mechanism of bladder cancer progression. A recent study has shown that the adaptor protein RacGAP1 inactivated GTP-bound Rac1 in bladder cancer, but the activation was inhibited by protein SHCBP1 (SHC-binding protein 1), which is a regulator of EGF (epidermal growth factor). Via this relay, SHCBP1 can inactivate Rac1 and promote bladder cancer progression [83]. The EGF/EGFR ligand/receptor couple is frequently overexpressed in bladder cancers, with squamous bladder cancers qualified as being EGFR-addicted [84]. This trend encourages the use of EGFR-targeted drugs to treat these cancers [85,86]. EGFR signaling generally follows the PAK1/Rac1 route to convey the signal and to regulate tumor progression [87,88]. EGF is known to stimulate both Rac1 and Pak1 in vascular smooth muscle cells (Figure 5) [89]. It is therefore possible to slow down the proliferation and migration of bladder cancer cells via a brake on Rac1. This can be done directly with Rac1 inhibitors or indirectly with molecules capable of controlling Rac1 expression and function. This is the case, for example, for the microRNA miR-142-3p, which interacts directly with Rac1 in bladder cancer cells to inhibit their proliferation but also their migration and invasion [90].

### 4.3. Rac1 in Epithelial-Mesenchymal Transition (EMT) of Bladder Cancer Cells

EMT is a biological process through which epithelial cells lose their epithelial phenotype and gain mesenchymal features. This process reflects the aggressive and invasive character of the tumor and is often correlated with metastasis. EMT is vital for the progression of bladder cancer tumors because it plays a crucial role in cancer cells spreading and invasion [91]. Numerous signaling proteins contribute to this cellular differentiation process and Rac1 is one of them [92]. The targeting of the EGFR-Rac1 axis can permit to reverse EMT [93]. The activation of Rac1 in the frame of the EMT implicates various modulating proteins, such as SPAG9 (sperm-associated antigen 9) and HEF1 (human enhancer of filamentation 1), which are both connected to Rac1 expression [94]. Other factors are implicated in Rac1-mediated EMT, such as the metabolic enzyme AKR1C1 (aldo-keto reductase 1C1), which mediates the invasive potential and drug resistance of metastatic bladder cancer cells (Figure 5). The inhibition of AKR1C1 reduces the invasion/metastatic potential of bladder cancer cells via the regulation of the Rac1/Src/Akt pathway and modulation of the production of inflammatory cytokines, such as interleukin 1β (IL-1β) [95]. The link between EMT and Rac1 has been studied more deeply in other types of cancers, notably in lung and colon cancers [96,97]. The contribution of Rac1 to the inflammation process shall not be neglected. Different studies have pointed out a marked reduction in the production of inflammatory cytokines upon the inhibition of the activation of Rac1, directly or through intermediate effectors [98,99,100]. Rac1 is a major actor of the crosstalk between the inflammatory state and tumor cell migration [101]. The expression and activation of Rac1 has frequently been found to enhance the production of pro-inflammatory cytokines (IL-1β, but also IL-6, IL-8 and TNFα) in different pathological situations [102,103]. For example, the Rac1-GEF interaction inhibitor 1D-142 reduces the nuclear translocation of the transcription factor NFκB induced by the cytokine TNFα in NSCLC cells, and this activity contributes significantly to the antitumor effect of this guanidine-type Rac1 inhibitor in vivo [104]. Rac1 can interact directly with specific cytokines, such as IL-37, which controls the membrane translocation of the protein and its signaling activities, at least in lung adenocarcinoma [105]. In this context, more attention should be paid to the alternatively spliced isoform Rac1B, the expression of which can be induced by pro-inflammatory extracellular signals in polarized colorectal cancer cells [106,107]. Rac1 protects cells from undergoing EMT in pancreatic and breast epithelial cells. Similar studies should be conducted with bladder cancer cells.

### 4.4. Rac1 in Bladder Cancer Metastasis

The role of Rac1 activation in tumor metastasis has been amply discussed, notably in the frame of various solid tumor types [28,46,108]. A comparable situation can be underlined in bladder cancer. Rac1 is a major player of the metastasis of bladder cancer [109]. The invasion and migration of bladder cancer cells depend, to some extent, on the activation status of Rac1 and the activity of its regulators, notably the aforementioned Rac1-binding protein aldoketo reductase 1C1 (AKR1C1), up-regulated in metastatic human bladder cancer specimens. AKR1C1 antagonists, such as the anti-inflammatory drug flufenamic acid, can be used to decrease the invasion potential of metastatic bladder cell lines [95]. A high activity of GTP-bound Rac1 (coupled with high expression of Pak1) has been measured in the lymph node metastasis of urothelial carcinoma of the upper urinary tract, thus providing a potential prognostic marker for this disease, but also reinforcing the idea that targeting Rac1 can reduce dissemination of the tumor [110].

Rac1 plays roles in tumorigenesis, tumor progression, EMT and metastasis of bladder cancer cells. The GTPase has been also implicated in other hallmarks of cancer, notably in stemness, immune escape and drug resistance [30,111]. These aspects have not been significantly studied in the frame of bladder cancer. For these reasons, we will not discuss further these aspects here, but they provide additional indirect lines of evidence supporting the interest of targeting activated Rac1 in bladder cancer.

The management of bladder cancer is excessively complex and variable, depending on the tumor types and stages. Endoscopic transurethral resection of bladder tumor represents the standard of care for non-muscle invasive bladder cancer. However, for more advanced bladder cancers, chemotherapy is required. Immunotherapeutic strategies for bladder cancers have also been largely developed in recent years through the use of immune checkpoint inhibitors (antibodies), adoptive cell therapy, cytokine-based therapy and antibody–drug conjugates [112]. However, there is always a need for new drugs and combinations to improve treatment efficacy and patients’ survival.

## 5. Small Molecules Targeting Rac1 to Treat Bladder Pathologies

**Figure 6:**
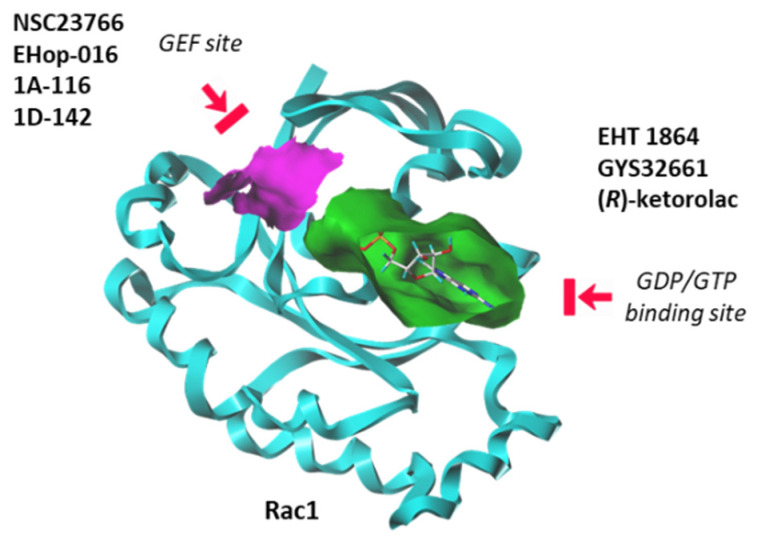
Two categories of Rac1 inhibitors. Some compounds fit into the GTP/GDP-binding site (in green) whereas other compounds bind to the GEF-binding site (in purple) and function as interrupters of Rac1-GEF interactions.

Until recently, Rho GTPases were deemed somewhat undruggable due to the promiscuity of the GDP/GTP binding pocket and the complexity of their regulatory mechanisms [113]. However, over the past seven years, significant progress has been made in deciphering the structural and dynamic properties of these GTPases. The use of NMR and other analytical methods has greatly helped to better comprehend the dynamic of Rac1 and its capacity to switch between active and inactive states [114]. Molecular modeling can also facilitate the identification and design of Rho GTPases inhibitors [115]. There exists now a panoply of Rac1-targeted small molecules, more or less specific to Rac1, and more or less potent at inhibiting GTP/GDP exchange or at modulating the interaction between Rac1 and GEF/GAP effectors [113,116]. These inhibitors can be separated in two groups: (i) compounds that block the interaction between Rac1 and GEF proteins, and (ii) molecules that bind to the GDP/GTP binding site of Rac1 in a GEF-independent manner. There are several molecules in both categories, as mentioned in Figure 6. Here, we will focus on a single representative of each group: the prototypic Rac1 inhibitor NSC23766, considered a blocker of the GEF-pocket of Rac1, and the thioquinoline derivative EHT 1864, which binds primarily to the nucleotide binding pocket, thus blocking Rac1 activation. Both compounds have been used and studied in the context of bladder pathologies.

### 5.1. GEF-Targeted Rac1 Inhibitor NSC23766 in Bladder Pathologies

**Figure 7:**
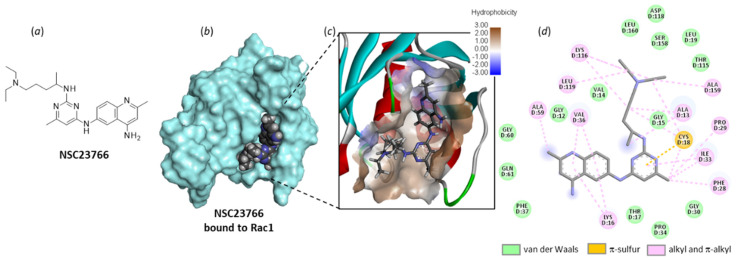
Molecular docking model of NSC23766 bound to Rac1 (PDB: 1I4D). (**a**) Chemical structure of NSC23766. (**b**) The compound bound to the protein surface (*cyan*). (**c**) A close-up view of NSC23766 binding site, with the hydrophilic/hydrophobic surface colored (indicated scale). (**d**) The binding map contacts for NSC23766 bound to Rac1. Calculated empirical energy of interaction ΔE = −58.0 kcal/mol and empirical energy of hydration ΔG = −23.9 kcal/mol.

NSC23766 was identified a long time ago as a Rac-specific small-molecule inhibitor [117]. The compound is moderately efficient at inhibiting Rac1 in cells (IC_50_ = 95.0 µM in MDA-MB-435 cells), but it represents a useful laboratory tool to block lamellipodial protrusion and cell migration in various cell types [118]. In human bladder smooth muscle cells, NSC23766 was found to decrease cell proliferation, and the effect was associated with a reduced expression of phospho-Rac1, as well as a repressed phosphorylation of MEK1/2 and ERK1/2, as observed with a Rac1 siRNA [69]. NSC23766 functions as a blocker of the interaction between Rac1 and GEF proteins, such as Trio and Tiam1 (T-lymphoma invasion and metastasis factor 1). A recent analysis of the binding of NSC23766 to the Rac1-Tiam1 interface has identified the key residues involved in the interaction, notably D38, N39, Y64 and L67 [119]. Our own docking analysis, performed with a different interface (the Rac1-p21 complex (PDB: 1I4D)), showed that the same amino acid sequences of Rac1 are implicated in the interaction with NSC23766. In our case, residues N39, Y64 and L67 were important, but also, several adjacent residues such as N57 and L70 were implicated in H-bonding interaction with the small molecule (Figure 7). The local configuration may slightly vary from one GEF to another, but globally, the binding zone remains the same.

NSC23766 is a *bona fide* Rac1 inhibitor, but its selectivity has been questioned. The compound has been shown to act as a competitive antagonist at muscarinic acetylcholine receptors, in addition to its Rac1 inhibitory properties [120]. It has been shown to also antagonize *N*-methyl-D-aspartate (NMDA) receptors in neurons of rodent species [121]. The compound is very useful at the research level to block Rac1 signaling implicated in the activation of smooth muscle contraction [70], notably to reduce the contractile activity in detrusor smooth muscle in diabetic rats [68]. Recently, in a model of isolated organ, NSC23766 was found to markedly inhibit detrusor contractions, competitively antagonizing muscarinic receptors [122]. It is a robust pharmacological tool, but in terms of drug development, more potent and more specific small molecules targeting Rac1 should be developed to reduce off-target effects and the risk of unwanted toxicities.

### 5.2. GTP-Antagonizing Rac1 Inhibitor EHT 1864 in Bladder Pathologies

**Figure 8:**
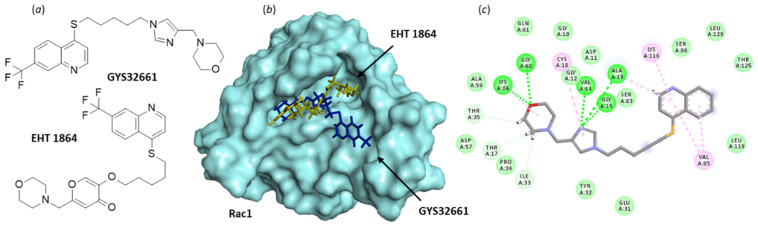
Binding of EHT 1864 and GYS32661 to Rac1. (**a**) Chemical structure of NSC23766. (**b**) Model of Rac1 (molecular surface, PDB: 1MH1) interacting with EHT 1864 (in yellow) and GYS32661 (in blue). The two molecules are superimposed to illustrate their distinct orientation within the binding site. (**c**) The binding map contacts for GYS32661 bound to Rac1. Calculated empirical energy of interaction ΔE = −143.1 and −74.7 kcal/mol and empirical energy of hydration ΔG = −25.8 and 15.7 kcal/mol, for GYS32661 and EHT 1864, respectively.

The second small molecule discussed here, EHT 1864 (Figure 8), is a potent inhibitor of Rac1-dependent lamellipodia formation in cells. It binds well to all Rac isoforms, Rac1 (and Rac1b), Rac2, and Rac3 (K_D_ = 40–60 nM) to inhibit Rac downstream signaling [123,124]. As such, the compound has been largely studied for its capacity to reduce growth of a variety of cell types, including smooth muscle cells and cancer cells, but investigations using cells with a bladder origin are rare. Like NSC23766, EHT 1864 can inhibit contractions of isolated detrusor muscle tissue, but unlike NSC23766, EHT 1864 does not competitively antagonize muscarinic receptors [122]. The compound has been found to inhibit detrusor contractions induced by the selective agonist of prostaglandin H2 (PGH2)/thromboxane A2 (TxA2) (TP) receptor agonist U46619, whereas NSC23766 was inefficient in this system [122]. Thus, both compounds can regulate detrusor smooth muscle contractions, but they apparently exhibit a divergent mechanism of action.

The pharmacological profile of EHT 1864 may be better adapted than that of NSC23766 to develop a Rac1-targeting drug. Analogs have been made, such as the imidazole derivative GYS32661 (developed by Revere Pharmaceuticals (Figure 8)) endowed with potent anticancer properties. In GYS32661, the g-pyrone (or 4-pyranone) moiety of EHT 1864 has been replaced with an imidazole ring. This structural modification considerably reinforces the Rac1-binding capacity of the compound. The docking analysis indicated that GYS32661 is much better adapted for binding to the GTP/GDP site of Rac1 than its parent compound EHT 1864 (Figure 8). The calculated empirical energy of interaction (ΔE) values reached −74.7 and −143.1 kcal/mol for EHT 1864 and GYS32661, respectively (with the PDB structure 1MH1 of Rac1). GYS32661 is currently developed as an anticancer agent, essentially positioned to treat breast and colon cancers [125], but this potent Rac1 inhibitor could be envisioned for the treatment of other malignancies associated with an overactivation of Rac1, such as bladder cancers.

There is no doubt that both EHT 1864 and NSC23766 are valid Rac1 inhibitors, but they target a different binding area of Rac1 and exhibit divergent effects in some cell systems. Whether this is due to the mode of interaction with Rac1 and the modulation of the signaling pathway, or due to off-target effects, remains to be determined. For example, an off-target effect on the chemokine receptor CXCR4 has been identified with NSC23766 [126], and at high concentration (100 mM), both EHT 1864 and NSC23766 can exert critical off-target effects on platelet functions, at least in a murine model [127]. Nevertheless, both compounds can be used to reduce growth and actin organization of bladder smooth muscle cells [128]. Rac1 is a valid target in several bladder pathologies, and there is now clear evidence that the protein is not “undruggable”. A few chemical series of Rac1-targeting small molecules have been proposed. There is room for the screening and design of novel compounds targeting this GTPase. Potent Rac1 inhibitors with nanomolar affinity for the protein and a high selectivity, such as those cited in recent publications and patents [30,129], can be considered for further development.

## 6. Rac1 Outside Bladder

The present review is centered around the role of Rac1 in bladder pathologies and the pharmacological targeting of the protein. However, evidently, the GTPase plays roles well beyond bladder pathologies and outside the genitourinary district. The protein is implicated in a large variety of pathologies, from various types of cancers [30,31] to neurodevelopmental disorders [128], to cite only two categories. Rac1 contributes to the regulation of blood pressure and the pathogenesis of pulmonary hypertension [25]. The protein is implicated in asthma-associated airway remodeling [130] and other pathophysiological processes. The focus on bladder presented here shall not underestimate the potential benefit of targeting Rac1 in other pathologies.

## 7. Conclusions and Perspectives

In cells, the GTP/GDP-bound dynamic cycle of Rac1 is like a crowded roundabout used by many signaling factors and implicated in multiple cellular functions. For these reasons, it is not surprising that this Rho GTPase is now viewed as a potential target to treat various oncologic and non-oncologic diseases [30]. The list of pathologies addressable with Rac1 inhibitors has expanded significantly in recent years to include diabetes, neurodevelopmental disorders, pulmonary hypertension, asthma and other pathologies [25,128,129,130,131]. In cancer, Rac1 is receiving more and more attention given the large implications of the protein in metastasis, drug resistance and immune modulation [43,46,132]. Our analysis indicates that Rac1 plays a significant role in several bladder dysfunctions and can be considered a target in three pathologic situations: bacterial infections of the bladder, diabetes-induced bladder diseases and bladder cancer. These pathologies can be interconnected. As mentioned above, diabetes mellitus is a risk factor for bladder cancer prognosis [64], and urinary incontinence is a common complication of bladder cancer [133].

Different Rac1 antagonists have been reported, such as EHT 1864 and NSC23766 evoked here, and a few other molecules. To our knowledge, two compounds are currently in preclinical development for the treatment of cancers: the imidazole derivative GYS32661 (Revere Pharmaceuticals, Boston, MA, USA) and the dual Cdc42/Rac inhibitor MBQ-167 (MBQ Pharma, Puerto Rico, US). The latter compound has been shown to inhibit Rac1/2/3 in MDA-MB-231 triple-negative breast cancer cells (IC_50_ = 103 nM) in addition to inhibiting the other GTPase Cdc42 (cell division control protein 42) (IC_50_ = 78 nM) [134]. It is a potent anticancer agent, at least at the preclinical level, currently positioned to treat triple-negative breast cancer [135,136]. However, the scope of tumors addressable with such a pan-Rac inhibitor is large and includes breast, colon, liver, lung, and other tumor types [132]. In this context, it would be interesting to consider further chemo-resistant prostate cancer, at least for two reasons. First, because Rac1 is usually overactivated in prostate cancer and Rac1 inhibition has been shown to reverse chemoresistance [137,138,139]. Second, because prostate cancer is frequently associated with bladder deficiency and lower urinary tract symptoms (LUTS). The prevalence of OAB symptoms has been found to be higher in patients receiving brachytherapy (internal radiation therapy) for prostate cancer compared to other treatment modalities [140]. LUTS and OAB are common in men with localized prostate cancer undergoing radical prostatectomy [141,142]. There is a link between prostatectomy and urinary bladder hypertrophy/dysfunctions [143]. Additionally, in women, the prevalence of urinary symptoms (OAB, urinary incontinence) is high in breast cancer patients treated with oral hormone therapy [144]. Moreover, urinary incontinence and OAB rates were found to be higher after gynecologic cancer surgery than in the general female population [145]. For these different reasons, the use of Rac1 inhibitors in cancer-associated bladder pathologies would make sense. A Rac1 antagonist, preferably those directly targeting the GDP/GTP binding site, could be an interesting option to treat advanced chemoresistance prostate cancer while reducing the risk of associated bladder dysfunction. This could be conducted via a chemotherapy regimen associating a Rac1 inhibitor and an evaluation of the bladder dysfunction symptom score. There are methods and tools to evaluate this score [146].

In summary, the present analysis highlights the interest and feasibility of targeting Rac1 to combat bladder pathologies, both non-oncologic bladder diseases such as OAB and bladder cancers. Multiple roles for Rac1 in bladder diseases have been evidenced in recent years. There is no doubt that this GTPase contributes to the dynamic of bladder muscle cells and bladder physiology in general. Targeting this Rho GTPase is a challenge, but doors have been opened with the design of selective inhibitors. Rac1 antagonists should experience a bright development in the coming years, and hopefully, patients will benefit from these advances in the near future.

## Data Availability

Not applicable.
